# Molecular Epidemiology of *Anaplasma capra* in Cattle from the Black Sea Region of Türkiye

**DOI:** 10.3390/pathogens15101084

**Published:** 2026-10-15

**Authors:** Husnu Furkan Sakar, Kursat Altay, Ufuk Erol, Omer Faruk Sahin

**Affiliations:** 1 Department of Parasitology, Institute of Health Sciences, Sivas Cumhuriyet University, 58140 Sivas, Türkiye; hfsakar@gmail.com; 2 Department of Parasitology, Faculty of Veterinary Medicine, Sivas Cumhuriyet University, 58140 Sivas, Türkiye; ufukerol@cumhuriyet.edu.tr (U.E.); ofsahin@cumhuriyet.edu.tr (O.F.S.)

**Keywords:** *Anaplasma capra*, TaqMan qPCR, nested PCR, *groEL*, cattle, Black Sea Region

## Abstract

*Anaplasma capra* is an emerging tick-borne pathogen with zoonotic potential. Since its first description, the pathogen has been reported in several countries across Asia, Europe, and Africa; however, information on its distribution in Türkiye remains limited. This study investigated its molecular occurrence in 654 cattle from 157 farms across six provinces in the Black Sea Region of Türkiye. DNA samples were examined using nested PCR and a previously developed TaqMan quantitative PCR assay targeting the *groEL* gene. Six samples tested positive by both methods, whereas ten additional samples were positive only by qPCR, resulting in an apparent molecular detection frequency of 2.45% (16/654). The paired assay results differed significantly (exact McNemar test, *p* = 0.00195), with moderate agreement between the methods (Cohen’s κ = 0.539; 95% CI: 0.288–0.791). Sequencing of the six nestedPCR-positive amplicons showed that all sequences were identical within the analyzed fragment and clustered within the known genotype 1 lineage. No significant associations were found between qPCR positivity and province, age, or sex. These findings provide molecular evidence of *A. capra* in cattle from the Black Sea Region. However, because the ten qPCR-only results were not independently confirmed, the estimated detection frequency and the potential value of qPCR for surveillance should be interpreted cautiously and validated using independent reference methods.

## 1. Introduction

Tick-borne diseases (TBDs) are among the most important vector-borne infections affecting humans and animals worldwide [1,2]. In recent years, changes in climate, environmental conditions, pasture use, animal movements, and host-vector interactions have influenced tick populations and the prevalence and epidemiological significance of the pathogens they transmit [1,2]. Tick-borne pathogens cause significant economic losses in livestock production due to reduced milk and meat yields, reproductive losses, increased treatment costs, and higher mortality rates [3,4]. Species of the genus *Anaplasma* are important tick-borne pathogens. Several species within this genus can infect domestic and wild animals as well as humans, causing anaplasmosis, an important concern for veterinary medicine and public health [2,4,5].

Advances in molecular diagnostic techniques have improved the detection and identification of *Anaplasma* species, leading to the identification of novel species within the genus [6]. One such species is *Anaplasma capra*, which was first detected in goats in China in 2012 and was subsequently recognized as a zoonotic pathogen following the report of human infections in 2015 [6,7]. Since its first description, *A. capra* has been detected in many vertebrate hosts, including domestic and wild animals and humans, as well as in various tick species in Asia, Europe, and Africa [8,9,10,11,12,13,14,15,16,17,18,19]. These findings indicate that the pathogen can infect a wide range of hosts and occur in different ecological settings, highlighting its importance in veterinary medicine and as a zoonotic pathogen [19].

Molecular identification of *A. capra* is primarily based on PCR amplification and sequencing of the *16S rRNA*, *gltA*, *groEL*, *MSP2*, and *MSP4* genes [16,18,20]. Among these genes, phylogenetic analyses of *gltA* and *groEL* have revealed two distinct genetic lineages, generally referred to as genotype 1 and 2 [10,20,21]. However, the *16S rRNA* gene is widely used for pathogen identification because it is highly conserved. However, its limited sequence variation makes it difficult to distinguish between *A. capra* genotype 1 and genotype 2 [20]. In contrast, the *groEL* gene shows greater nucleotide variation, providing higher phylogenetic resolution for characterizing genetic diversity within the species and distinguishing between the two genotypes [7,10,20,21].

Conventional and nested PCR assays are widely used for the detection of *A. capra*. However, quantitative PCR (qPCR) offers several advantages for molecular detection, including high analytical sensitivity and specificity, detection of low numbers of target DNA, reduced risk of contamination due to its closed-tube system, and shorter time to results [22,23,24]. These features make qPCR a suitable method for epidemiological studies involving large numbers of field samples. A *groEL*-targeted TaqMan qPCR assay for *A. capra* was recently developed and analytically evaluated by Altay et al. [24], together with conventional and colorimetric LAMP assays [24]. Altay et al. [24] showed that the TaqMan qPCR assay performed better than nested PCR and could detect *A. capra* DNA at lower concentrations than the nested PCR assay [25,26].

Türkiye is divided into seven geographical regions characterized by distinct climatic and geographical features. Among these regions, the Black Sea Region is an important livestock-producing area due to its favorable climatic and ecological conditions, which may also support tick development and survival [27]. High rainfall, persistent humidity, dense vegetation, and widespread pasture-based livestock farming may provide favorable conditions for tick survival and distribution. These conditions may also provide a suitable environment for the presence and transmission of tick-borne pathogens in the region [27]. These ecological conditions suggest that the region may provide a suitable environment for the spread of *A. capra*. However, no study has investigated the presence of *A. capra* in cattle in the Black Sea Region of Türkiye. This highlights a significant gap in the data regarding the geographic distribution and epidemiology of *A. capra* in Türkiye.

Accordingly, this study aimed primarily to investigate the occurrence and epidemiological distribution of *A. capra* in cattle from six provinces of the Black Sea Region of Türkiye. A secondary objective was to characterize the *A. capra* isolates detected in nested PCR-positive samples by *groEL*-based sequence and phylogenetic analyses. The established TaqMan qPCR assay was used alongside nested PCR to support molecular detection and to describe differences in assay-based detection patterns in the newly collected regional dataset.

## 2. Material and Methods

### 2.1. Study Area and Samples

**Figure 1:**
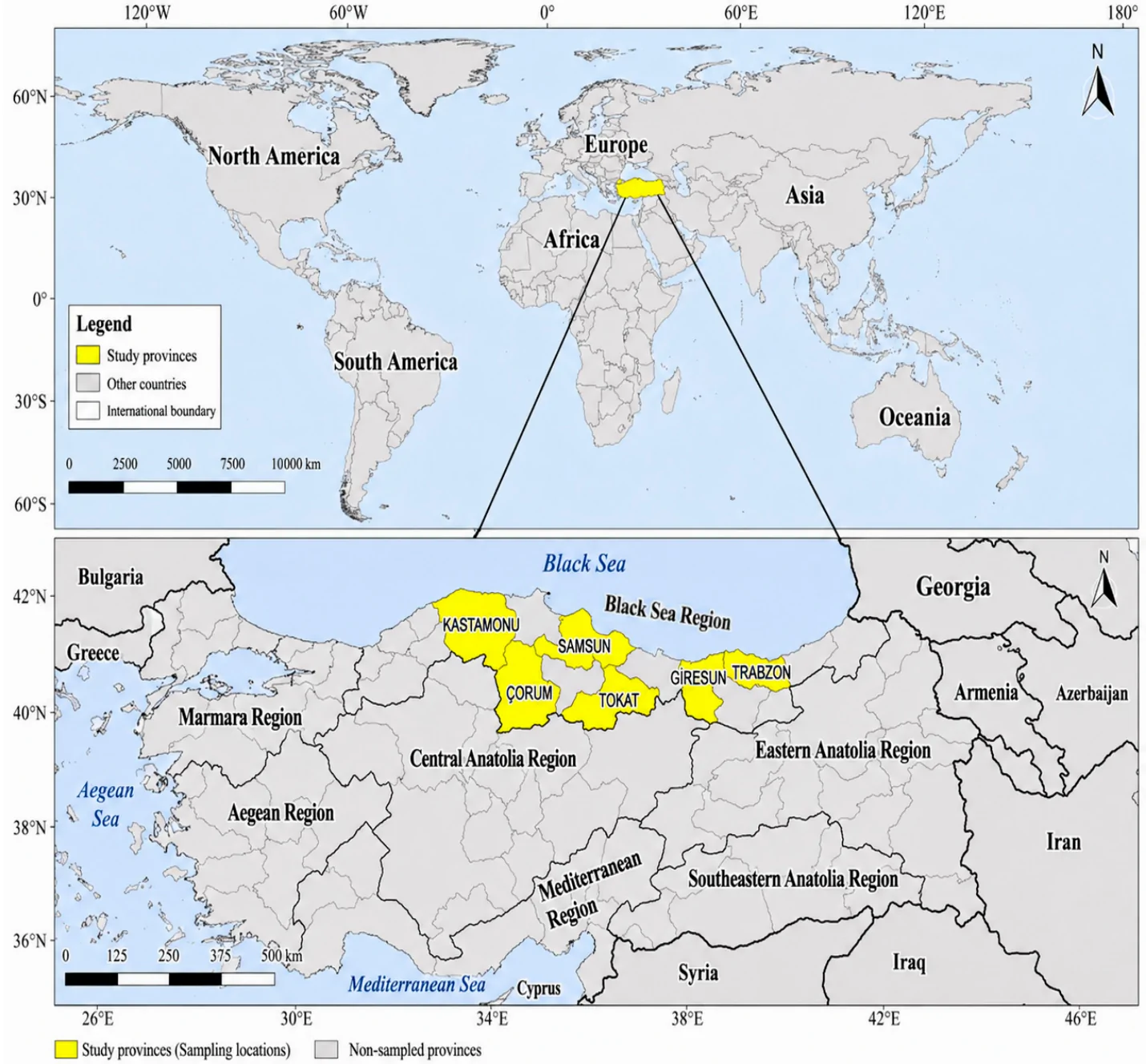
Location of Türkiye and sampling areas in different parts of Türkiye. The map was generated using QGIS version 4.2.

Blood samples were collected from cattle in six provinces of the Black Sea Region of Türkiye: Samsun, Çorum, Tokat, Kastamonu, Giresun, and Trabzon (Figure 1, Table 1). The study area encompasses two major climatic zones. The coastal provinces of Giresun, Trabzon, Samsun, and Kastamonu are characterized by a humid climate, with high rainfall throughout the year and generally mild temperatures. In contrast, the inland provinces of Tokat and Çorum have continental and semi-arid climatic characteristics, with hotter summers, colder winters, and greater seasonal temperature variations.

The 654 cattle blood samples, including 494 females and 160 males, were newly collected between April 2023 and July 2024 from 157 cattle farms in Samsun, Çorum, Tokat, Kastamonu, Giresun, and Trabzon. The distribution of the samples by province and district, sampling dates, number of farms, number of samples, and molecular detection results is provided in Supplementary Table S1. The 25 cattle samples included in our previous publication [24] were collected in Sivas Province. In contrast, all samples analyzed in the present study were collected from the six provinces listed above, and there was no overlap in the sampling areas between the two studies. Therefore, the cattle samples analyzed in the present study were newly collected and geographically distinct from those reported previously [24]. The animals ranged in age from 8 months to 10 years and were all raised under pasture-based production systems. All cattle were clinically healthy at the time of blood sampling. After being delivered to the laboratory, the samples were stored at −20 °C until genomic DNA extraction.

### 2.2. DNA Extraction and Molecular Detection of Anaplasma capra by Nested PCR and TaqMan qPCR

Genomic DNA (gDNA) was extracted from whole-blood samples using the PureLink™ Genomic DNA Kit (Cat. No. K1820–02; Invitrogen™, Carlsbad, CA, USA) according to the manufacturer’s instructions. Throughout the DNA extraction procedure, positive—*A. capra* (GenBank accession no. ON783820; [20])—and negative—DNase-/RNase-free sterile water (Qiagen^®^, Hilden, Germany)—controls were included to avoid false positive and negative results. The extracted gDNA was stored at −20 °C until molecular analysis.

Initially, 654 DNA samples were screened for *A. capra* using a nested PCR assay. The primer sets used for nested PCR are listed in Table 2. In the first-round PCR, a 1129-bp fragment of the *groEL* gene was amplified as previously described by Li et al. [7]. The resulting amplicons were then subjected to nested PCR to generate an 874-bp fragment of the same gene, following the protocol of Yang et al. [28]. Each PCR was performed in a final volume of 25 µL. The first-round PCR mixture contained 14.375 µL of sterile distilled water (Cat. No. 129114, Qiagen^®^, Germany), 2.5 µL of 10× PCR buffer (Thermo Scientific™, Vilnius, Lithuania), 2.5 µL of 25 mM MgCl_2_ (Thermo Scientific™, Vilnius, Lithuania), 200 µM of each dNTP (Cat. No. R0181, Thermo Scientific™, Vilnius, Lithuania), 1.25 U of Taq DNA polymerase (Cat. No. EP0402, Thermo Scientific™, Vilnius, Lithuania), 1 µL of each outer primer (10 pmol/µL), and 3 µL of template DNA. For the nested PCR, the reaction mixture was prepared in a final volume of 25 µL and contained 15.875 µL of sterile distilled water (Cat. No. 129114, Qiagen^®^, Hilden, Germany), 2.5 µL of 10× PCR buffer (Thermo Scientific™, Vilnius, Lithuania), 2.5 µL of 25 mM MgCl_2_ (Thermo Scientific™, Vilnius, Lithuania), 200 µM of each dNTP (Cat. No. R0181, Thermo Scientific™, Vilnius, Lithuania), 1.25 U of Taq DNA polymerase (Cat. No. EP0402, Thermo Scientific™, Vilnius, Lithuania), 1 µL of each inner primer (10 pmol/µL), and 1.5 µL of template DNA. The *A. capra*-positive DNA sample (GenBank accession no. ON783820) was used as the positive control, whereas DNase/RNase-free sterile water (Cat. No. 129114, Qiagen^®^, Hilden, Germany) was used as the negative control. Amplified products were separated by electrophoresis on 1.6% agarose gels and visualized under ultraviolet (UV) illumination. To minimize the risk of contamination, DNA extraction, PCR preparation, and post-amplification assays were carried out in physically separated laboratory areas.

In this study, the above-mentioned 654 gDNA samples were also tested using the previously established *groEL*-based TaqMan qPCR assay [24]. The *A. capra groEL*-specific TaqMan qPCR assay was performed using a BlasTaq™ Probe 2× qPCR MasterMix (abm^®^, Richmond, BC, Canada). Reaction mixtures were prepared according to the manufacturer’s recommendations, with the forward and reverse primer concentrations adjusted to 300 nM each and the hydrolysis probe concentration to 150 nM. DNA extracted from an *A. capra* positive sample (GenBank accession no. ON783820) was included as the positive control, while DNase-/RNase-free water (Cat. No. 129114, Qiagen, Hilden, Germany) served as the no-template control (NTC).

Amplification was carried out using a Q1000+ Real-Time PCR System (LongGene, Hangzhou, China). Cycling conditions consisted of an initial enzyme activation step at 95 °C for 3 min, followed by 40 cycles of denaturation at 95 °C for 15 s and annealing/extension at 58 °C for 1 min. The assay targeted an 89-bp region of the *groEL* gene using the primer–probe set shown in Table 3 [24]. Samples with quantification cycle (Cq) values ≤ 35 were considered positive for *A. capra*.

### 2.3. Sequencing and Phylogenetic Analysis

The *groEL* gene was sequenced in all nested PCR-positive samples. Before sequencing, positive amplicons were excised from agarose gels and purified using a commercial gel extraction kit (PCR Clean-Up & Gel Extraction Kit, GeneDireX^®^, Taoyuan City, Taiwan, Cat. No. NA006-0300); all procedures were performed according to the manufacturer’s protocol. The purified products were subsequently subjected to bidirectional sequencing using the BigDye Terminator v3.1 Cycle Sequencing Kit and analyzed on an ABI 3730XL Genetic Analyzer (Applied Biosystems, Foster City, CA, USA). The quality of the resulting sequence chromatograms was assessed using FinchTV version 1.4.0 software (Geospiza Inc., Seattle, WA, USA). Consensus sequences were generated and compared with reference sequences available in the GenBank database using the MUSCLE alignment algorithm implemented in MEGA-X [29].

The resulting consensus sequences were submitted to GenBank, and accession numbers were assigned to each sequence. The phylogenetic analyses were performed using the *groEL* sequences obtained in this study and reference *A. capra* sequences retrieved from GenBank. A phylogenetic tree was constructed using the Maximum Likelihood (ML) method in MEGA-X [29], with branch support assessed using 1000 bootstrap replicates. Based on the Best-Fit Substitution Model analysis implemented in MEGA-X, the Kimura-2 parameter model with gamma distribution and invariant sites (G + I) was identified as the most appropriate evolutionary model for ML analysis [30]. The phylogenetic tree was subsequently constructed using this model.

### 2.4. Statistical Analysis

Statistical analyses were performed to evaluate differences in the prevalence of *A. capra* infection among the sampled provinces, age groups, and sexes, and to compare the detection results obtained by nested PCR and TaqMan qPCR. Descriptive data were expressed as numbers, percentages, and 95% confidence intervals (CIs), which were calculated using the Wilson score method. Because both molecular assays were performed on the same 654 blood samples, the results were considered paired binary observations. A 2 × 2 contingency table was constructed, and differences between the two paired detection methods were assessed using the exact McNemar test. Fisher’s exact test was used to assess associations between *A. capra* detection and province, age group, and sex. Agreement between the two assays was assessed using Cohen’s kappa coefficient (κ) with a 95% confidence interval. A two-sided *p*-value < 0.05 was considered statistically significant. The Cq values of the concordant (nPCR+/qPCR+) and discordant (nPCR−/qPCR+) samples were compared using the Mann–Whitney U test. Because the comparison involved small, outcome-defined groups, this analysis was considered exploratory.

## 3. Results

### 3.1. Detection of Anaplasma capra by Nested PCR and TaqMan qPCR

**Figure 2:**
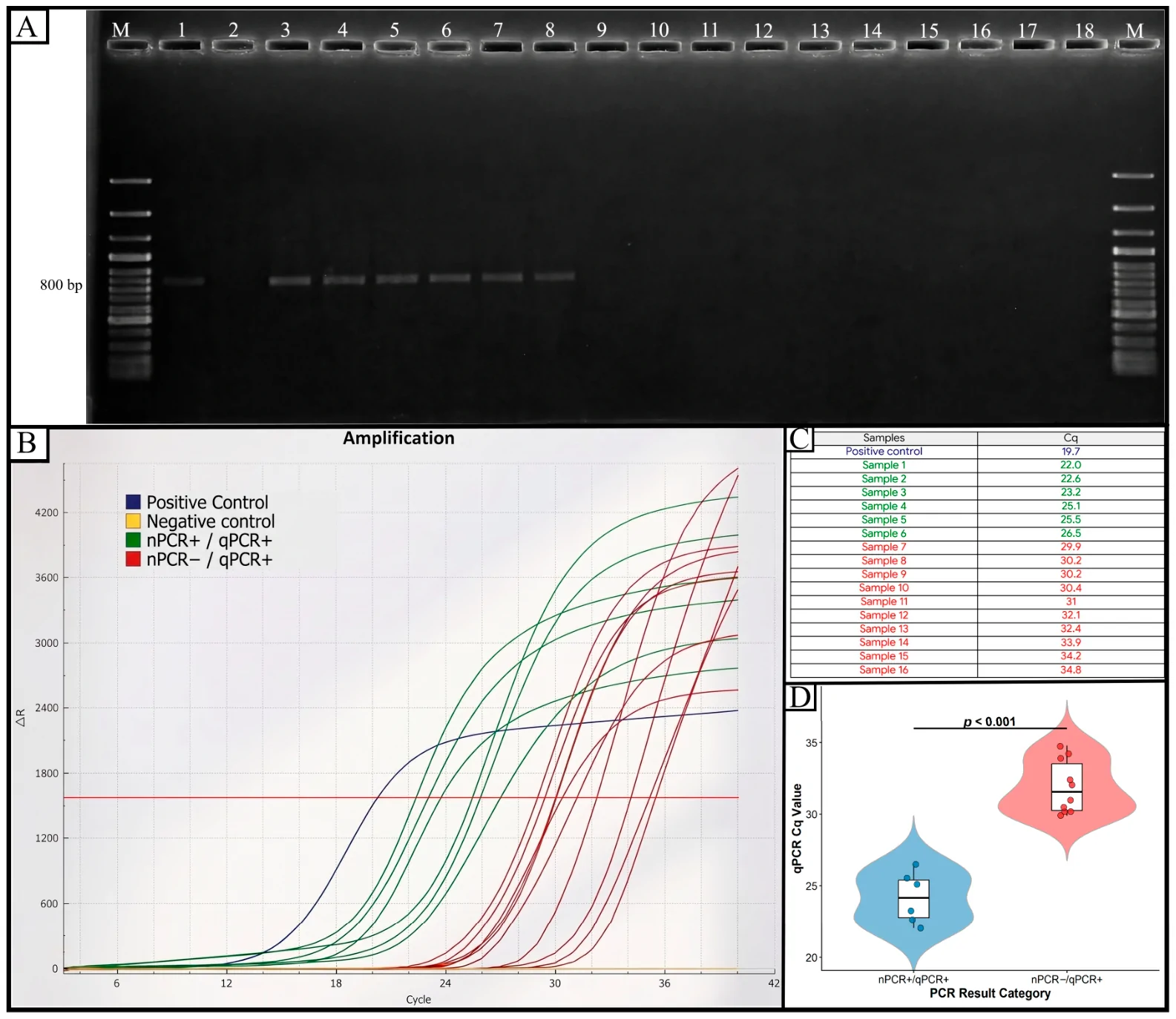
Molecular detection of *A. capra*. (**A**) Agarose gel electrophoresis of the *groEL* nested PCR products. **M.** Marker; **1**. *A. capra* positive control; **2.** Negative control; **3–8.** Nested PCR-positive samples; **9–18.** Nested PCR-negative samples. (**B**) Amplification curves obtained by TaqMan qPCR. (**C**) Individual Cq values of the 16 TaqMan qPCR-positive samples. Samples 1–6 were positive by both nested PCR and TaqMan qPCR, whereas samples 7–16 were positive only by TaqMan qPCR. (**D**) A violin plot showing the Cq values for concordant (nPCR+/qPCR+) and discordant (nPCR−/qPCR+) samples. The *p*-value was obtained using the Mann–Whitney U test. nPCR+/qPCR+: positive by nested PCR and TaqMan qPCR; nPCR−/qPCR+: negative by nested PCR and positive by TaqMan qPCR.

A total of 654 cattle blood samples collected from the Black Sea Region of Türkiye were tested for *A. capra* DNA using nested PCR and TaqMan qPCR. *Anaplasma capra* DNA was detected by nested PCR in six samples (0.92%; 95% CI: 0.42–1.99%), whereas 16 samples were positive by TaqMan qPCR (2.45%; 95% CI: 1.50–3.96%) (Figure 2). The 16 TaqMan qPCR-positive samples originated from 13 farms (Supplementary Table S1). Six samples were positive by both assays, while ten samples were positive only by TaqMan qPCR. The ten qPCR-positive/nested PCR-negative samples had Cq values ranging from 29.9 to 34.8. These findings represent assay-positive results rather than independently confirmed infections (Figure 2) (Table 4).

The distribution of assay-positive samples varied among the sampled provinces. Using nested PCR, *A. capra* DNA was detected in Samsun (4/148, 2.70%; 95% CI: 1.05–6.74%), Kastamonu (1/118, 0.85%; 95% CI: 0.15–4.72%), and Giresun (1/117, 0.85%; 95% CI: 0.15–4.76%), whereas no nested PCR-positive samples were identified in Çorum, Tokat, or Trabzon. TaqMan qPCR yielded positive results in all six provinces, including Samsun (7/148, 4.73%; 95% CI: 2.31–9.43%), Giresun (3/117, 2.56%; 95% CI: 0.88–7.24%), Kastamonu (2/118, 1.69%; 95% CI: 0.47–5.98%), Çorum (2/89, 2.25%; 95% CI: 0.62–7.85%), Tokat (1/102, 0.98%; 95% CI: 0.17–5.35%), and Trabzon (1/80, 1.25%; 95% CI: 0.22–6.76%).

Among the 16 TaqMan qPCR-positive samples, six were also positive by nested PCR, whereas ten were negative by nested PCR (Table 4). The Cq values of the ten discordant qPCR-positive samples ranged from 29.9 to 34.8 (Figure 2). Because these samples were not independently confirmed using another molecular target, sequencing, repeat extraction, or an alternative assay, the qPCR-only results were reported as assay-positive findings and were not interpreted as confirmed infections.

The nested PCR and TaqMan qPCR results were evaluated by age group and sex (Table 5). No nested PCR-positive samples were detected in cattle younger than one year (n = 89) or older than five years (n = 174). All six animals that tested positive by nested PCR were 2–4 years old, with a prevalence of 1.53% in this age group (6/391; 95% CI: 0.70–3.30%). According to sex, all nested PCR-positive samples were obtained from female cattle, with a prevalence of 1.21% (6/494; 95% CI: 0.56–2.61%). No nested PCR-positive samples were detected among male cattle (0/160). Using TaqMan qPCR, *A. capra* DNA was detected in 2 of 89 cattle younger than one year (2.25%; 95% CI: 0.62–7.85%), 9 of 391 cattle aged 2–4 years (2.30%; 95% CI: 1.22–4.31%), and 5 of 174 cattle older than five years (2.87%; 95% CI: 1.23–6.53%). In addition, TaqMan qPCR detected *A. capra* DNA in 12 of 494 female cattle (2.43%; 95% CI: 1.39–4.21%) and 4 of 160 male cattle (2.50%; 95% CI: 0.98–6.27%) (Table 5).

No statistically significant association was found between *A. capra* DNA detection and age group, sex, or province (*p* > 0.05). Among the 654 paired samples, six were positive by both nested PCR and TaqMan qPCR, whereas ten were positive only by TaqMan qPCR. No samples were positive by nested PCR and negative by TaqMan qPCR, while 638 were negative by both methods (Table 4). The exact McNemar test showed a statistically significant difference between the paired detection results (*p* = 0.00195). The overall agreement between nested PCR and TaqMan qPCR was 98.47%; Cohen’s kappa coefficient was 0.539 (95% CI: 0.288–0.791), indicating moderate agreement between the two assays.

### 3.2. Phylogenetic Analysis

The partial sequences of the *groEL* gene were successfully obtained from all nested PCR-positive samples. Following sequence alignment, consensus sequences were generated and deposited in GenBank under accession numbers PX432044–PX432049.

Sequence comparison showed that the six *A. capra* sequences obtained in this study shared 100% nucleotide identity with each other. BLAST analysis (NCBI,) showed that the sequences had 90.91–100% nucleotide similarity with *A. capra* sequences available in GenBank. The highest similarity (100%) was observed with isolates previously reported from a human (OQ819448) and a water buffalo (ON783820) in Türkiye, as well as an isolate obtained from sheep in France (MW930531). The lowest similarity (90.91%) was observed with a goat isolate from China (MH174931).

**Figure 3:**
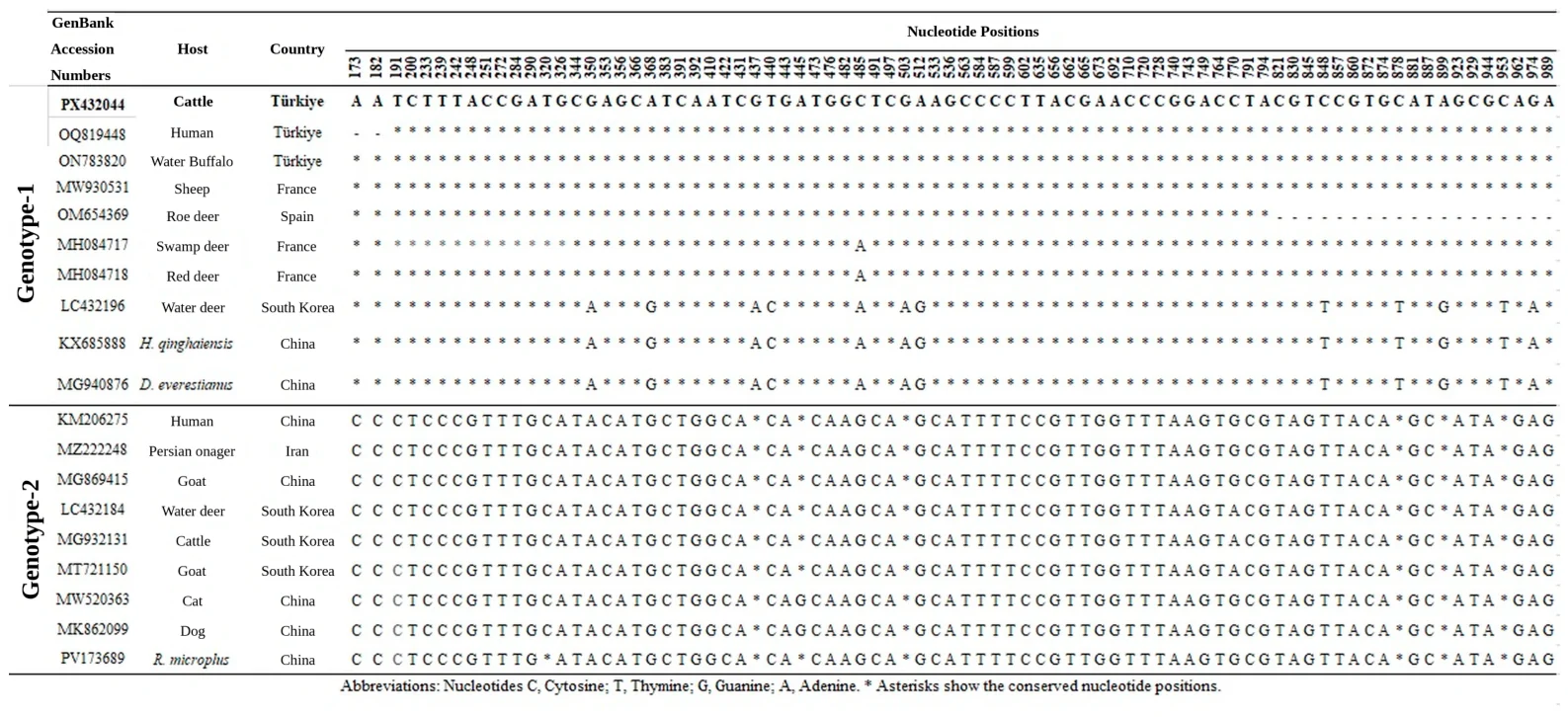
Nucleotide differences table according to partial sequences of the *A. capra groEL* gene.

Detailed phylogenetic analyses of the *groEL* gene sequence revealed that single nucleotide polymorphisms (SNPs) were present between the sequences obtained in this study and the isolates from swamp deer (MH084717) and red deer (MH084718) reported from France. Twelve nucleotide differences were identified relative to water deer isolates from South Korea (LC432196) and *Haemaphysalis qinghaiensis* (KX685888) and *Dermacentor everestianus* (MG940876) isolates from China. In addition, comparisons of our *A. capra* isolates and *A. capra* isolates obtained from humans (KM206275), goats (MG869415 and MT721150), cats (MW520363), dogs (MK862099), *Rhipicephalus microplus* (PV173689), Asiatic wild asses (MZ222248), cattle (MG932131), and water deer (LC432184) revealed 73–75 nucleotide differences (Figure 3).

**Figure 4:**
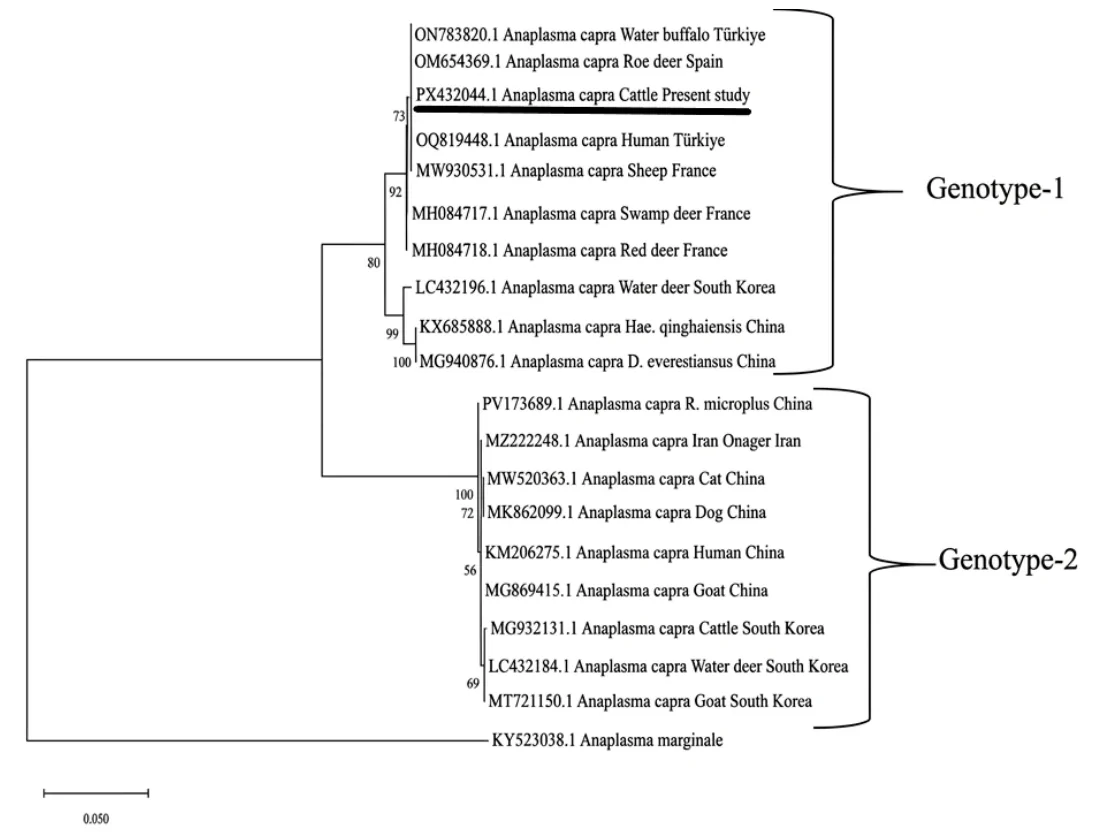
Phylogenetic tree based on the *groEL* gene sequences of *A. capra* isolates using the maximum likelihood method. Numbers at the nodes represent the bootstrap values with 1000 replicates. The evolutionary history was inferred using the Maximum Likelihood method and the Kimura 2-parameter model [30]. *Anaplasma marginale* was used as an outgroup in the phylogenetic trees. Evolutionary analyses were conducted in MEGA-X [29].

Phylogenetic relationships were determined using the Maximum Likelihood (ML) method based on partial *groEL* gene sequences of *A. capra* and representative sequences obtained from GenBank (Figure 4). Phylogenetic tree analysis based on partial *groEL* gene sequences revealed that *A. capra* isolates were divided into two genotypes.

All *A. capra* isolates identified in the present study clustered within the genotype 1 clade together with previously reported reference isolates. Node support was evaluated using 1000 bootstrap replicates. *Anaplasma marginale* was included as the outgroup, and phylogenetic reconstruction was performed in MEGA-X using the Maximum Likelihood method under the Kimura 2-parameter substitution model.

## 4. Discussion

Tick-borne pathogens are a significant problem in veterinary and public health because they can infect humans as well as domestic and wild animals. Among these pathogens, *Anaplasma* species are becoming increasingly significant due to their wide geographic distribution and their ability to infect a variety of hosts [4,7,20]. Among the newly identified species in this group, *A. capra* has been detected in livestock, wild animals, ticks, and humans in many countries across Asia, Europe, and Africa [9,10,11,13,16,17,18,19,31,32,33,34,35,36]. Although this pathogen has been detected in a wide range of hosts worldwide, research on *A. capra* in Türkiye remains limited. In addition, no information is currently available on the presence of this pathogen in the Black Sea Region. Therefore, this study provides the first molecular evidence of *A. capra* in cattle from the Black Sea Region and contributes to a better understanding of its geographic distribution and phylogenetic features in Türkiye.

In clinically healthy hosts, the pathogen load is generally low, and the pathogen may be unevenly distributed in peripheral blood. Consequently, *A. capra* may remain undetected because of its low density in peripheral blood. However, clinical signs may develop as the infection progresses or when the host’s immune system is weakened, potentially leading to production losses [7,11,37]. Therefore, accurate detection of *A. capra* infection in hosts is important for appropriate treatment and for developing effective measures to control and prevent the infection. For this reason, molecular methods are essential for reliable diagnosis and epidemiological investigations [19,24]. Conventional and nested PCR assays are commonly used for the detection of *A. capra*. However, infections with a low bacterial load may not always be detected, particularly under field conditions [24]. Quantitative PCR assays can reduce the risk of contamination through closed-tube systems and facilitate the detection of low bacterial loads. In this study, the presence of *A. capra* was investigated using both nested PCR and a TaqMan qPCR assay targeting the *groEL* gene. The findings provide molecular evidence of *A. capra* in cattle from the Black Sea Region and also allowed a direct comparison of the two molecular methods.

Differences in *A. capra* prevalence have been reported among cattle populations from different regions [19]. The prevalence of *A. capra* in cattle has been reported to vary between 0.28% and 11.3% across different countries [19]. In the present study, *A. capra* was detected in 0.92% of cattle by nested PCR and in 2.45% by TaqMan qPCR. The prevalence determined in this study was within a range similar to that reported in previous studies. Compared with the prevalence detected by TaqMan qPCR in the present study, lower prevalence was reported in South Korea (0.3–0.4%) [25,38], Kyrgyzstan (0.28%) [18], and Malaysia (1.4%) [39]. In contrast, higher infection rates were reported in China (5.6%) [40], Angola (6.2%) [41], Morocco (11.3%) [42], and Kyrgyzstan (3.01%) [43]. In addition, the number of studies on the presence and prevalence of *A. capra* in Türkiye has increased in recent years [16,20,44]. However, substantial gaps remain in our understanding of the epidemiology of *A. capra* in animal hosts in Türkiye. The available studies show that this pathogen occurs in different livestock species, but its prevalence is generally low. The first molecular detection of *A. capra* in cattle was reported by Altay et al. [16], with a prevalence of 0.41% in cattle from Sivas using a *gltA*-based nested PCR assay. Subsequent studies detected the pathogen in water buffaloes (14.28%) from the same region [20] and in goats (0.5%) from Van [44], indicating that *A. capra* has been detected in different host species across Türkiye. Although the Black Sea Region provides suitable ecological conditions for tick survival due to its climate, dense vegetation, and widespread pasture-based livestock farming, the prevalence observed in this study was relatively low. Similar findings have been reported from several endemic regions where *A. capra* was detected at a low prevalence despite the presence of vectors [25,33]. These differences may be related to several factors, including climate, vegetation, tick populations in sampling areas, livestock management practices, host populations, sampling strategies, and molecular methods used for pathogen detection. Therefore, prevalence rates between studies should be compared with caution [45,46,47,48,49]. Seasonal variation in tick activity should be considered when interpreting the prevalence observed in this study. In the present study, samples were collected from April 2023 to July 2024, covering spring and summer periods in two consecutive years. However, tick abundance and activity were not directly monitored; therefore, the effect of specific tick activity peaks on *A. capra* prevalence could not be determined. As all cattle were maintained under pasture-based systems, seasonal exposure to ticks may have contributed to pathogen transmission. Furthermore, less sensitive diagnostic methods may fail to detect infections with low pathogen loads.

The sensitivity, specificity, and detection limits of molecular-based methods may vary [24]. In this study, the apparent molecular detection frequency was 0.92% by nested PCR and 2.45% by TaqMan qPCR. TaqMan qPCR yielded positive results in 16 samples, compared with six positive samples by nested PCR. The paired analysis showed a significant difference between the two methods, with ten samples testing positive only by TaqMan qPCR and none testing positive only by nested PCR. However, these findings should be interpreted in terms of assay-based molecular detection rather than diagnostic sensitivity because an independent reference standard was not available. Therefore, the higher number of qPCR-positive samples does not, by itself, establish superior diagnostic sensitivity. The ten qPCR-positive/nested-PCR-negative samples had Cq values ranging from 29.9 to 34.8, whereas the six samples positive by both assays had lower Cq values ranging from 22.0 to 26.5. This pattern may be consistent with lower target DNA levels in the qPCR-only samples and may partly reflect the previously demonstrated analytical performance of the TaqMan qPCR assay [24]. In contrast, the higher analytical sensitivity of TaqMan qPCR can improve infection detection by facilitating the identification of DNA targets present in low copy numbers; indeed, it has been reported that TaqMan qPCR detects a greater number of positive samples compared to conventional PCR methods in the molecular diagnosis of various tick-borne pathogens and offers an advantage in identifying low-level infections [22,23,24,50]. However, Cq values alone do not establish infection, and the ten qPCR-only results were not independently confirmed by repeat extraction, an alternative molecular target, sequencing, or another independent assay. Therefore, these results are reported strictly as assay-positive findings and were not considered confirmed infections. Accordingly, the 2.45% value based on all 16 qPCR-positive samples should be interpreted as an apparent molecular detection frequency rather than a confirmed infection prevalence, whereas the six nested-PCR-positive samples represent the sequence-confirmed subset. In this study, no significant association was found between *A. capra* infection and age, sex, or geographical origin. Similar findings have been reported in several molecular epidemiological studies, with no consistent associations reported between *A. capra* infection and age, sex, or geographical origin [18,25,40]. The lack of significant differences in this study may be explained by the relatively low number of positive animals and the inclusion of clinically healthy cattle raised under similar pasture-based management systems, with similar levels of exposure to vectors [18,25,40]. In addition, the low prevalence found in this study may have limited the statistical power to detect possible associations between infection and host-related factors.

DNA sequence analysis is an important method for assessing the genetic diversity of pathogens. In this context, epidemiological findings provide information on the prevalence and distribution of *A. capra* in the Black Sea Region, while sequence analysis provides further information on the genetic characteristics of the circulating isolates [11,51]. In this study, all *groEL* sequences obtained from cattle were identical. This finding indicates a high level of genetic conservation within the analyzed gene fragment and suggests limited genetic diversity among the *A. capra* isolates obtained from cattle in the study area. However, further studies involving different hosts, vectors, and geographical regions are needed to better understand the overall genetic diversity of *A. capra* in Türkiye. BLAST analysis also showed that the isolates identified in this study were identical to previously reported isolates from humans and water buffaloes in Türkiye and were closely related to isolates detected in sheep in France. These findings suggest that the *A. capra* isolates identified in the Black Sea Region are genetically related to isolates previously reported from different host species and geographical regions. In contrast, the lower sequence similarities with some Asian isolates reflect the genetic diversity previously reported for *A. capra*. Overall, these results support the use of the *groEL* gene as a reliable molecular marker for investigating the genetic relationships and geographical distribution of *A. capra* isolates. The close genetic relationship among the isolates was also supported by the phylogenetic analysis [52,53,54]. Similar to previous studies, the *groEL*-based phylogenetic tree divided *A. capra* into two main genotypes [10,20,21]. All isolates identified in this study clustered within genotype 1 together with reference sequences previously reported from different hosts and geographical regions. This finding indicates that the *A. capra* isolates identified in cattle in the Black Sea Region belong to genotype 1, which has also been reported in Türkiye and Europe. Previous studies have suggested that genotype distribution may be influenced by both geographical origin and host species, but the relationship between these factors is not yet fully understood [10,20]. In this study, only genotype 1 was detected, which is consistent with previous studies from Türkiye. This finding also supports the current view that this genotype is the predominant genotype circulating in the country. However, the limited number of sequenced isolates and the lack of samples from wildlife and ticks prevent a complete assessment of the genetic diversity of *A. capra* in the Black Sea Region. Future studies including a larger number of host species, vectors, and wider datasets will provide further information on the epidemiology and transmission dynamics of this emerging pathogen.

This study has some limitations that should be considered when interpreting the findings. First, the qPCR-positive/nested PCR-negative samples were not independently confirmed; therefore, their infection status could not be established with certainty, and the higher number of qPCR-positive samples should not be interpreted as evidence of superior diagnostic sensitivity. Second, tick abundance and seasonal activity were not directly monitored, which limited our ability to assess the effect of tick activity on *A. capra* prevalence. Third, the study was limited to cattle from six provinces in the Black Sea Region, and the findings may not represent the prevalence of *A. capra* in other regions or host species in Türkiye. Another limitation is the clustered sampling design, as multiple cattle were sampled from the same farms. Although farm-level sampling was considered in the interpretation, the small number of positive animals prevented reliable multilevel modelling. Therefore, the reported confidence intervals and associations may not fully account for within-farm correlation and should be interpreted cautiously. Finally, although the TaqMan qPCR assay used in this study was previously described, the present study applied this method to a new and larger cattle sample set to provide epidemiological data on *A. capra* in the study area. Further studies including independent confirmation of discordant samples, direct assessment of tick activity, and broader geographical and host sampling are warranted.

## 5. Conclusions

This study provides the first molecular evidence of *A. capra* in cattle from the Black Sea Region of Türkiye and expands the known geographical distribution of the pathogen in the country. TaqMan qPCR assay-positive results were obtained in all six sampled provinces, whereas sequence-confirmed detections were obtained from three provinces. No significant association was observed between assay-based detection and province, age, or sex. The six sequenced isolates were identical within the analyzed *groEL* fragment and clustered within genotype 1, indicating that the detected cattle isolates belong to a previously recognized lineage. The comparison of nested PCR and TaqMan qPCR showed different assay-based detection patterns, including ten qPCR-positive/nested PCR-negative results; however, these discordant results were not independently confirmed and therefore cannot be used to establish superior diagnostic sensitivity. TaqMan qPCR may be useful for molecular surveillance, particularly for samples with low target DNA levels, but this potential should be evaluated further using independent reference methods and confirmation of discordant results. The detection of *A. capra* in cattle is also relevant to public health because this pathogen has been reported as a tick-borne zoonotic agent in humans, highlighting the importance of continued surveillance at the animal–tick–human interface. Continued epidemiological surveillance involving cattle, other hosts, ticks, and seasonal sampling will be important for clarifying the distribution and transmission of *A. capra* in Türkiye.

## Figures and Tables

**Table 1:** Distribution of blood samples by province.

Location	Number of Animals
Samsun	148
Kastamonu	118
Giresun	117
Trabzon	80
Çorum	89
Tokat	102
**Total**	**654**

**Table 2:** Primers used for amplification of the *Anaplasma capra groEL* gene.

Target Gene	Primer Name	Primer Sequence (5′-3′)	Amplicon Size (bp)	Reference
*groEL*	Outer-f	GCGAGGCGTTAGACAAGTCCATT	1129	[7]
	Outer-r	TCCAGAGATGCAAGCGTGTATAG		
	Inner-f	TGAAGAGCATCAAACCCGAAG	874	[28]
	Inner-r	CTGCTCGTGATGCTATCGG		

**Table 3:** Primer and probe sequences used in the *groEL*-targeted TaqMan qPCR assay.

Names of Primers and Probe	Primer Sequence (5′-3′)	Reference
Acp TaqMan-F	GCAAAGGGTTCAAAGACCT(A/C)GA	[24]
Acp TaqMan-R	CTCAAACTCTACCAGCATCTTCTC	
Acp TaqMan-Probe	TGCAGTT(C/T)GATCGCGGATACCTTT	

**Table 4:** Cross-tabulation of paired nested PCR and TaqMan qPCR results for the detection of *Anaplasma capra* in 654 cattle blood samples.

Nested PCR	TaqMan qPCR Positive	TaqMan qPCR Negative	Total
Positive	6	0	6
Negative	10	638	648
**Total**	**16**	**638**	**654**

**Table 5:** Detection of *Anaplasma capra* by nested PCR and TaqMan qPCR according to province, age group, and sex, with exact *p*-values.

Variable	Number of Animals	Nested PCR, n (%) [95% CI]	*p*-Value	TaqMan qPCR, n (%) [95% CI]	*p*-Value
**Province**			**0.308**		**0.557**
Samsun	148	4 (2.70) [1.05–6.74]		7 (4.73) [2.31–9.43]	
Çorum	89	0		2 (2.25) [0.62–7.85]	
Tokat	102	0		1 (0.98) [0.17–5.35]	
Kastamonu	118	1 (0.85) [0.15–4.72]		2 (1.69) [0.47–5.98]	
Giresun	117	1 (0.85) [0.15–4.76]		3 (2.56) [0.88–7.24]	
Trabzon	80	0		1 (1.25) [0.22–6.76]	
**Age group**			**0.086**		**0.933**
≤1 year	89	0		2 (2.25) [0.62–7.85]	
2–4 years	391	6 (1.53) [0.70–3.30]		9 (2.30) [1.22–4.31]	
≥5 years	174	0		5 (2.87) [1.23–6.53]	
**Sex**			**0.344**		**1.000**
Female	494	6 (1.21) [0.56–2.61]		12 (2.43) [1.39–4.21]	
Male	160	0		4 (2.50) [0.98–6.27]	
**Total**	**654**	**6 (0.92) [0.42–1.99]**		**16 (2.45) [1.50–3.96]**	

Note: *p*-values for province, age group, and sex were calculated using Fisher’s exact test. A two-sided *p*-value < 0.05 was considered statistically significant.

## Data Availability

The data supporting the findings of this study are provided in the Supplementary Material. Supplementary Table S1 presents the distribution of the 654 cattle samples by province, district, sampling period, number of farms, and nested PCR and TaqMan qPCR detection results. Supplementary Table S2 provides sample-level information for the molecularly positive samples, including sample ID, province, district, age/age group, sex, sampling date or period, nested PCR result, TaqMan qPCR result, individual Cq value, and GenBank accession number where applicable. Individual animal and farm identifiers are not provided to preserve confidentiality. The six *groEL* sequences generated in this study are available in GenBank under accession numbers PX432044–PX432049.

## Supplementary Materials

The following supporting information can be downloaded at https://www.mdpi.com/article/10.3390/pathogens15101084/s1. Supplementary Table S1. Distribution of the 654 cattle samples by province and district, sampling period, number of farms, and molecular detection results. Supplementary Table S2. Dataset table of Nested PCR and qPCR positive samples.
