# Supplementary material for: Molecular Epidemiology of Anaplasma capra in Cattle from the Black Sea Region of Türkiye
Source: Pathogens. 2026 Oct 15;15(10):1084. doi: 10.3390/pathogens15101084 (PMC13708661; doi:10.3390/pathogens15101084)
Supplement: Supplementary file 1 [file pathogens-15-01084-s001.zip › pathogens-4573608-supplementary.pdf]

Supplementary Table S1. Distribution of the 654 cattle samples by province and district, sampling period, number of farms, and molecular detection results.

Province	District	Sampling date(s)	No. of farms	No. of samples	nPCR-positive (n)	qPCR-positive (n)
Trabzon	Yomra	June 2024	7	46	0	1
	Tonya	June 2024	3	23	0	0
	Vakfıkebir	June 2024	2	11	0	0
**Trabzon subtotal**			**12**	**80**	**0**	**1**
Samsun	Central	April 2023	2	26	0	1
	Tekkeköy	May 2023	8	45	0	1
	Kavak	June 2024	2	13	2	2
	Bafra	July 2024	4	29	0	1
	Vezirköprü	July 2024	1	5	0	0
	Terme	July 2024	1	30	2	2
**Samsun subtotal**			**18**	**148**	**4**	**7**
Kastamonu	Central	June 2024	10	33	0	1
	Tosya	June 2024	4	25	1	1
	Araç	June 2024	1	3	0	0
	Seydiler	June 2024	1	4	0	0
	Ağlı	June 2024	1	1	0	0
	Taşköprü	June 2024	1	1	0	0
	Devrekani	June 2024	3	12	0	0
	Daday	June 2024	4	11	0	0
	İhsangazi	June 2024	4	16	0	0
	İnebolu	June 2024	2	12	0	0
**Kastamonu subtotal**			**31**	**118**	**1**	**2**
Giresun	Central	April 2023	18	33	0	2
	Dereli	April 2023	6	15	0	0
	Şebinkarahisar	May 2023	12	36	1	1
	Çamoluk	May 2023	7	7	0	0
	Görele	July 2024	5	14	0	0
	Alucra	July 2024	5	12	0	0
**Giresun subtotal**			**53**	**117**	**1**	**3**
Tokat	Central	June 2023	1	3	0	0
	Artova	June 2023	9	29	0	0
	Turhal	June 2024	6	19	0	0
	Reşadiye	June 2024	5	14	0	1
	Almus	June 2024	4	12	0	0

Province	District	Sampling date(s)	No. of farms	No. of samples	nPCR-positive (n)	qPCR-positive (n)
	Pazar	June 2024	1	25	0	0
**Tokat subtotal**			**26**	**102**	**0**	**1**
Çorum	Central	May 2024	6	47	0	1
	Mecitözü	May 2024	3	11	0	0
	Alaca	July 2024	2	5	0	0
	Sungurlu	July 2024	2	6	0	0
	Dodurga	June 2024	1	3	0	1
	Bayat	June 2024	3	17	0	0
**Çorum subtotal**			**17**	**89**	**0**	**2**
**Grand total**	**6 provinces, 36 districts**		**157**	**654**	**6**	**16**

nPCR, nested polymerase chain reaction; qPCR, quantitative real-time polymerase chain reaction. "Central" refers to the central district of each province.

Supplementary Table S2. Dataset table of Nested PCR and qPCR positive samples.

Sample ID	Province	District	Age/Age group	Sex	Sampling period/date	nPCR-positive	qPCR-positive	Cq value	GenBank Accession
TRS 21	Trabzon	Yomra	1	M	June 2024	-	+	31	-
QS 108	Samsun	Central	6	F	April 2023	-	+	30.2	-
QS 24	Samsun	Tekkeköy	7	F	May 2023	-	+	29.9	-
QS 48	Samsun	Kavak	4	F	June 2024	+	+	22.6	PX432045
QS 56	Samsun	Kavak	4	F	June 2024	+	+	25.1	PX432046
QS 78	Samsun	Bafra	4	F	July 2024	-	+	32.1	-
QS 139	Samsun	Terme	2	F	July 2024	+	+	25.5	PX432047
QS 140	Samsun	Terme	4	F	July 2024	+	+	26.5	PX432048
YS 102	Kastamonu	Central	1	M	June 2024	-	+	30.2	-
YS 11	Kastamonu	Tosya	4	F	June 2024	+	+	23.2	PX432049
JS 6	Giresun	Central	5	M	April 2023	-	+	32.4	-
JS 17	Giresun	Central	3	F	April 2023	-	+	34.8	-
JS 101	Giresun	Şebinkarahisar	3	F	May 2023	+	+	22.0	PX432044
ÖS 55	Tokat	Reşadiye	6	F	June 2024	-	+	34.2	-
ÇRS 26	Çorum	Central	7	F	May 2024	-	+	30.4	-
ÇRS 67	Çorum	Dodurga	3	M	June 2024	-	+	33.9	-

nPCR, nested polymerase chain reaction; qPCR, quantitative real-time polymerase chain reaction. “Central” refers to the central district of each province. M: Male, F: Female.
